# Density Functional Theory and Molecular Docking Investigations of the Chemical and Antibacterial Activities for 1-(4-Hydroxyphenyl)-3-phenylprop-2-en-1-one

**DOI:** 10.3390/molecules26123631

**Published:** 2021-06-14

**Authors:** Ahmed M. Deghady, Rageh K. Hussein, Abdulrahman G. Alhamzani, Abeer Mera

**Affiliations:** 1 Basic Science Department, Higher Technological Institute, 10th of Ramadan City 44629, Egypt; am_deghady@yahoo.com; 2 Physics Department, College of Science, Imam Mohammad Ibn Saud Islamic University (IMSIU), Riyadh 11623, Saudi Arabia; 3 Chemistry Department, College of Science, Imam Mohammad Ibn Saud Islamic University (IMSIU), Riyadh 11623, Saudi Arabia; agalhamzani@imamu.edu.sa; 4 Physics Department, College of Arts and Science, Prince Sattam Bin Abdulaziz University, Wadi Addawasir 11991, Saudi Arabia; a.mera@psau.edu.sa; 5 Physics Department, Faculty of Science, Kafrelsheikh University, Kafrelsheikh 33516, Egypt

**Keywords:** DFT, HOMO-LUMO, MEP, FTIR, FT-Raman, molecular docking

## Abstract

The present investigation informs a descriptive study of 1-(4-Hydroxyphenyl) -3-phenylprop-2-en-1-one compound, by using density functional theory at B3LYP method with 6-311G** basis set. The oxygen atoms and π-system revealed a high chemical reactivity for the title compound as electron donor spots and active sites for an electrophilic attack. Quantum chemical parameters such as hardness (η), softness (S), electronegativity (χ), and electrophilicity (ω) were yielded as descriptors for the molecule’s chemical behavior. The optimized molecular structure was obtained, and the experimental data were matched with geometrical analysis values describing the molecule’s stable structure. The computed FT-IR and Raman vibrational frequencies were in good agreement with those observed experimentally. In a molecular docking study, the inhibitory potential of the studied molecule was evaluated against the penicillin-binding proteins of Staphylococcus aureus bacteria. The carbonyl group in the molecule was shown to play a significant role in antibacterial activity, four bonds were formed by the carbonyl group with the key protein of the bacteria (three favorable hydrogen bonds plus one van der Waals bond) out of six interactions. The strong antibacterial activity was also indicated by the calculated high binding energy (−7.40 kcal/mol).

## 1. Introduction

Chalcones are common metabolic compounds derived from nature, which attracted huge interest in several applications. Edible plants such as apples, vegetables, licorice, tea, and many other natural foods are valuable for chalcones. The molecular structure of chalcones defined as aromatic ketones in which three carbon α,β-unsaturated system (two carbons atoms connected to conjugated carbonyl group) formed a bridge between two aromatic rings [1,2]. Chalcone can form in two isomers known as either Cis- or Trans isomers due to the double bond in the structure. The varied content of beneficial biochemical in chalcones makes it related to many diseases such as atherosclerosis, cancer and Alzheimer’s disease [3,4]. Also chalcones are antifungal, antimicrobial, anti-inflammatory and antitumor properties due to their antioxidant effects [5,6,7]. A wide range of studies have been reported on chalcones, using chalcone as a privileged scaffold in medicinal chemistry has resulted in recent advances published in the past years [8,9,10]. The interesting electronic and nonlinear optical properties of chalcones derivatives act as a springboard towards exploring different and new applications.

**Figure 1:**
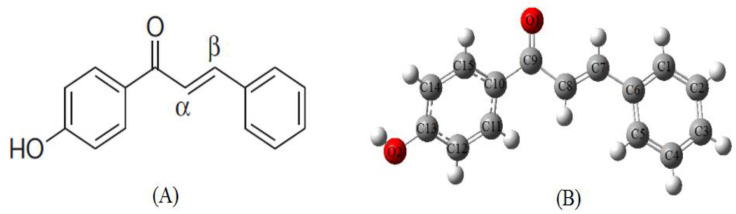
Chemical molecular structure (**A**) and optimized structure (**B**) for 1-(4-Hydroxyphenyl)-3-phenylprop-2-en-1-one.

1-(4-Hydroxyphenyl)-3-phenylprop-2-en-1-one is a well-known type of α, β- unsaturated chalcones. The title compound has an s-cis conformation with electron conjugation between the central chain and the attached rings as can be seen in Figure 1A. Classical and recent methods were used in the synthesis of 1-(4-Hydroxyphenyl)-3-phenylprop-2-en-1-one, including the use of different heteropolyacid catalysts and water as a green solvent [11,12]. Like many other types of chalcones, 1-(4-Hydroxyphenyl)-3-phenylprop-2-en-1-one has been integrated as starting materials in the synthesis of a set of heterocyclic compounds such as quinolinones, isoxazoles, thiadiazines, and benzofuranones [13,14]. There are still some ambiguities concerning the precise mechanisms of action for the different activities of chalcones. Therefore, the mentioned compound still in need of more qualitative and descriptive studies.

Theoretical calculations, such as the Density Functional Theory method (DFT), have emerged as a powerful technique for assessing the structural and spectral properties of organic compounds. Many DFT studies have been published describing a broad range of chalcones properties [15,16,17,18,19]. The characterization of synthesized novel chalcones by substitution groups in their derivative structures was also achieved using DFT [20,21,22]. Molecular docking is an effective strategy to gain insight into ligand-receptor interactions in the drug design industry. The vital role of molecular docking in the development of drug design is due to its ability to predict the best binding mode between drugs and the target protein. Molecular docking methods were used widely to analyze the biological and antibacterial activities of chalcones [23,24,25].

In the current work, an investigative study using DFT methods has been carried out in order to provide a deeper understanding of 1-(4-Hydroxyphenyl)-3-phenylprop-2-en-1-one compound (See Supplementary Materials). This includes the analysis of geometrical structure, electronic, spectroscopic properties, and chemical reactivity. For exploring the role of the carbonyl group in the antibacterial activities of chalcones, a molecular docking study was performed for the title molecule against one of the main proteins of Staphylococcus aureus bacteria (S. aureus). 

## 2. Results and Discussion

### 2.1. Vibrational Spectra Analysis

**Figure 2:**
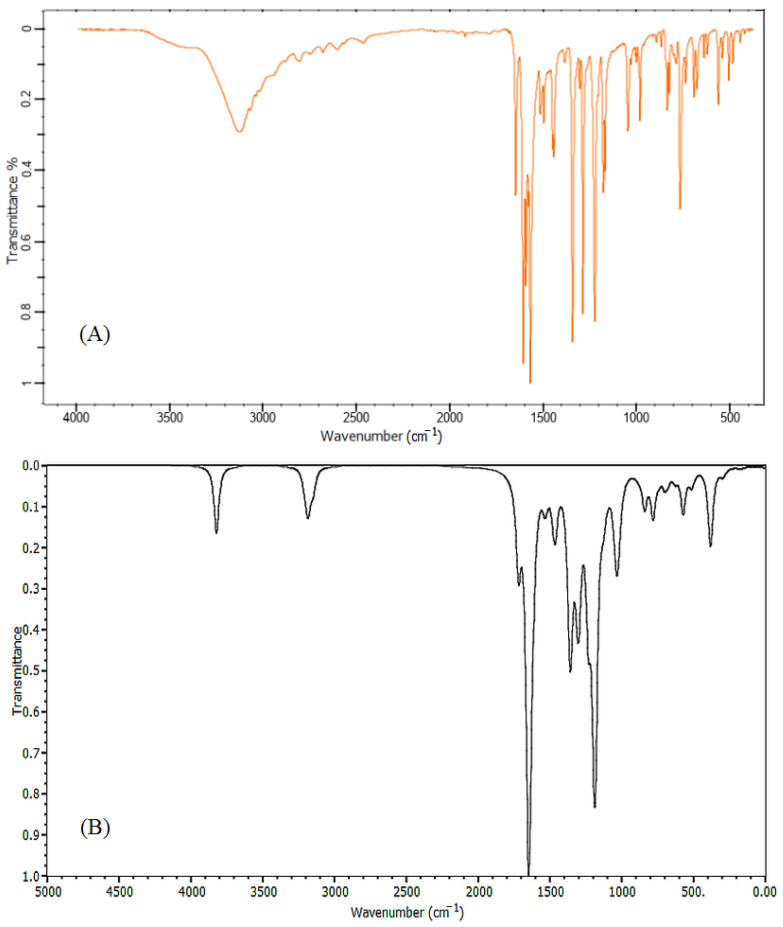
Experimental (**A**) and simulated (**B**) FT-IR spectra of 1-(4-Hydroxyphenyl)-3-phenylprop-2-en-1-one.

**Figure 3:**
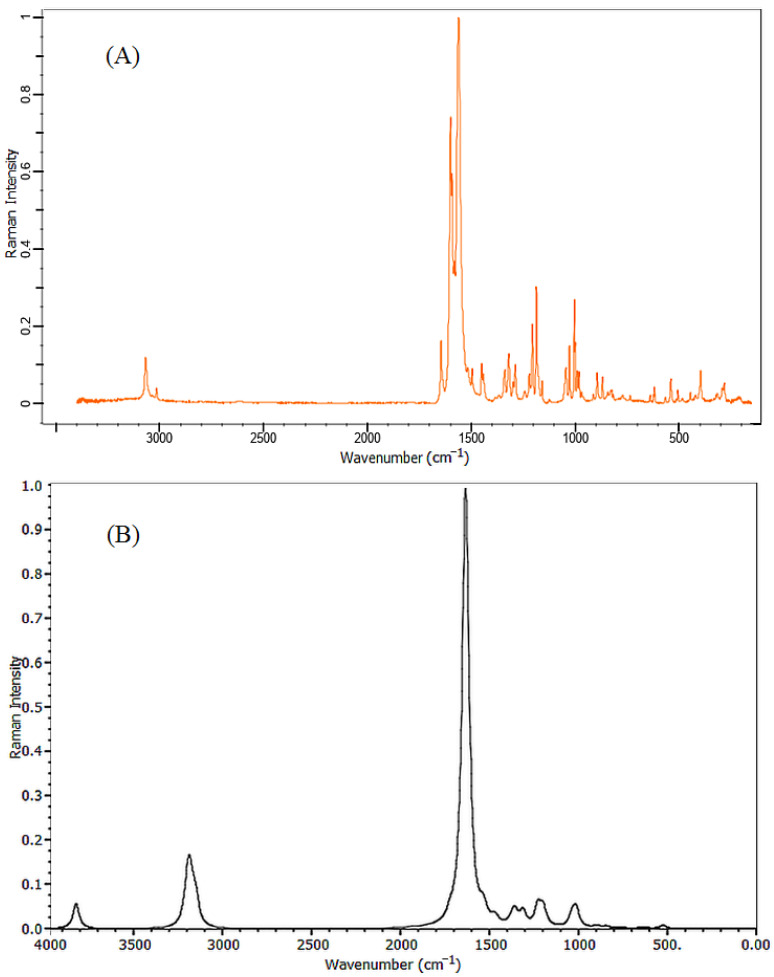
Experimental (**A**) and simulated (**B**) Raman spectra of 1-(4-Hydroxyphenyl)-3-phenylprop-2-en-1-one.

Figure 2 and Figure 3 show the calculated FT-IR and Raman vibrational spectra of the title compound along with experimental data. The characteristic bands were recorded in Table 1. The overestimation computing the vibrational frequencies was corrected by applying a suitable scaling factor, anharmonicity, and approximation in electronic structure calculations were corrected using the scaling factor 0.9899.

The conventional knowledge of the infrared spectrum intensities states that any band exceeds 3500 cm^−1^ is classified as an O–H stretching vibration [26]. In our DFT investigation, the stretching O–H vibration was located at 3787 cm^−1^. The C–H vibrational group has characteristic bands specified in the range of 3000 cm^−1^ to 3250 cm^−1^ and recognizable as C–H stretching vibrations [27]. In the obtained results, the C–H stretching band was observed at 3120 cm^−1^ and was calculated at 3152 cm^−1^. The spectrum of the carbonyl group show stretching vibration in the range of 1750–1680 cm^−1^, while the C=C group has stretched vibrations appearing in the region 1670–1620 cm^−1^ [28]. The calculated band at 1705 cm^−1^ is a stretching vibrational mode of the carbonyl group (C=O) and this peak was observed at 1648 cm^−1^. A strong band assigned at 1654 cm^−1^ describes the characteristic stretching vibrational mode for the C=C double bond and was found at 1594 cm^−1^ in the observed data. Two other bands, 1574 cm^−1^, and 1513 cm^−1^, were observed for the mixed mode of the C=O and C=C stretching vibrations, these vibrations were calculated at 1628 and 1620 cm^−1^ respectively. The aromatic ring demonstrated stretching vibration at 1606 and 1599 cm^−1^, these bands were observed at 1569 and 1551 cm^−1^ in the experimental data.

The range of 1300–1000 cm^−1^ is usually assigned to C–H in-plane bending vibrations [29]. In the present molecule, the C–H rocking vibration of the phenyl ring was calculated at 1350 cm^−1^ and was observed at 1333 cm^−1^. Moreover, C–H rocking vibration was assigned at1308 cm^−1^. In the IR spectrum, C–O has a stretching band that appears in the frequencies range from 1210 cm^−1^ to 1320 cm^−1^ [30]. In the present investigation, the C–O single bond vibration was specified at 1287 cm^−1^. This assignment value was extremely similar to the observed result 1281 cm^−1^. The C–H in-plane scissoring vibration of the phenol ring was assigned at 1176 cm^−1^, whereas its observed value was located at 1165 cm^−1^, other scissoring mode vibrations were assigned at 1172, 1118, and 1093 cm^−1^. The C–H out-of-plane bending (twisting vibrations) was observed at 978 cm^−1^ and calculated at 984 cm^−1^, a series of twisting vibrations bands were found at 970, 940, and 921 cm^−1^. A high intensity out plane bending vibration identified as C–H wagging mode was observed at 772 cm^−1^, this mode was assigned with low intensity at 776 cm^−1^ in results computed by BYLYP method.

In Raman spectra, C–H stretching vibration was assigned at 3060 cm^−1^. The stretching vibration of carbonyl group C=O was located at 1645 cm^−1^. The stretching mode of the double bond C=C was described by a prominent band at 1594 cm^−1^. The C=O and C=C mixed-mode stretching vibrations were specified at 1552 cm^−1^. The out-of-plane bending frequencies represented in rocking, scissoring, and twisting mode were observed at 1319, 1198, and 998 cm^−1^, respectively. Finally, it can be inferred that the investigated vibrational frequencies were within the expected regions and were in good agreement with both experimental and literature data.

### 2.2. Molecular Geometry

The optimized molecular structure was given in Figure 1B. A comparison of selected optimized geometric parameters with experimental data [31] is shown in Table 2. The experimental and calculated bond lengths are in good agreement with each other. The C-C bond length in the central chains C6-C7, C8-C9, C9-C10 are observed at 1.46, 1.47, 1.46 Å respectively, while the C-C bond distances in the two aromatic rings are found to be around the range1.39 Å due to the delocalization of electrons. The carbonyl double bond C9=O1 has a shorter bond length (1.23 Å) than carbon-oxygen single bond C13-O2 (1.36 Å). The characteristic torsion angle C7-C8-C9-O1 was found −11.37° which is very similar to the value obtained from experimental results −11.40°. The calculated torsion angles of C15-C10-C9-C8 (cal 154.22°, exp 155.73°), C10-C9-C8-C7 (Cal 167.42°, exp 168.50°) and C9-C8-C7-C6 (cal −176.58°, exp −177.17°) evidenced that the molecule is not planar.

### 2.3. HOMO and LUMO Analysis

**Figure 4:**
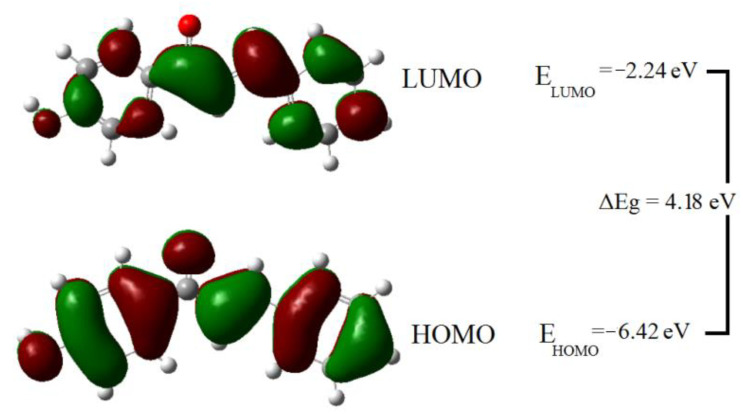
Surface plots showing the studied molecule’s frontier molecular orbitals.

The frontier molecular orbitals are known as the highest occupied molecular orbital (HOMO) and the lowest unoccupied molecular orbital (LUMO) are very important identifiers for electric properties in quantum chemistry. HOMO stands for electron donors while the LUMO represents electron acceptors, light absorption corresponds to the transition from HOMO orbitals to LUMO orbitals. Figure 4 depicted the surfaces of the frontier molecular orbitals for the mentioned compound. The HOMO and LUMO orbitals are clearly dispersed over the whole molecule, except for hydrogen atoms in HOMO and hydrogen atoms and carbonyl oxygen in LUMO. The contour surfaces of HOMO were found distributed basically over the π-system ring and C7=C8 bond, the accumulation of electron clouds on the oxygen atoms indicates that it participates in the charge transfer property. LUMO densities are located mainly on the carbon atoms of both the phenyl, phenol rings and on the C-C single bond of the central chain. Therefore, the HOMO to LUMO electronic transition is primarily obtained from the π-π* electronic transitions due to the presence of rich electron clouds of π atomic orbitals. 

### 2.4. Analysis of the Conceptual DFT Indices

The correlation between the calculated energies of molecules and their corresponding quantum parameters is used to provide details about the chemical activity. The quantum chemical parameters such as hardness (η), softness (S), electronegativity (χ), and electrophilicity (ω) are global descriptors for the chemical behavior of the molecules [32]. The hardness value determines how the atom resists the charge transfer to another atom or metal surface. The ability of an atom to receive electrons is measured by the softness value. Electronegativity χ is a chemical property that describes a molecule’s tendency to attract electrons. The scale of electrophilic property of a molecule is determined by the electrophilicity index ω. Table 3 shows the Koopmans theorem’s [33] definitions of these chemical descriptors, as well as their calculated values. The nucleophilicity index was measured according to Luis Domingo’s work in analyzing the reactivity of captodative ethylenes compounds [34]. Quantum chemical studies of equivalent chalcone derivatives to the studied molecule were performed previously. 1-(4-Hydroxyphenyl)-3-phenylprop-2-en-1-one was found to have lower hardness and higher softness value than those previously reported [35]. Furthermore, the studied molecule has a high electronegativity and electrophilicity index value. The nucleophilicity index value was found to be 2.95 eV, classifying the studied molecule as moderate nucleophiles [36]. The above findings support that the investigated molecule has a high chemical reactivity.

### 2.5. Molecular Electrostatic Potential (MEP)

**Figure 5:**
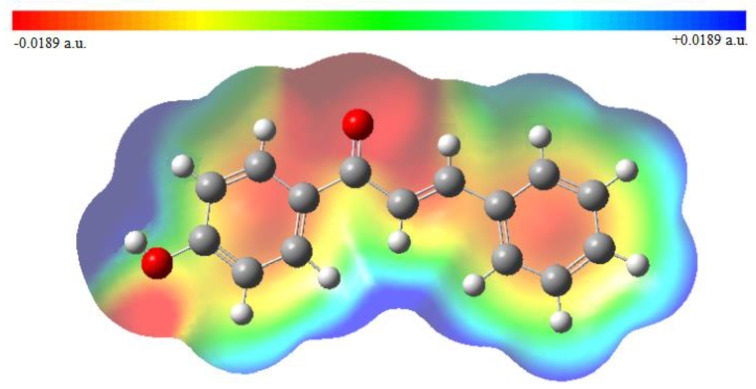
Molecular electrostatic potential surface for the studied molecule.

Molecular electrostatic potential (MEP) is a very important identifier for electrophilic and nucleophilic attacks based on the electrostatic potential distribution. The positive electrostatic spots are associated with the nucleophilic attack and negative electrostatic spots are preferable regions for an electrophilic attack. The electrostatic potential regions in MEP are visualized in terms of color-coding. The positive and negative electrostatic regions are represented by blue and red colors, respectively, whereas a zero potential region is specified by the green color. The positive electrostatic potential on the molecule surface is increasing in the direction red → yellow → green → light blue → blue. As shown in Figure 5, the most positive regions in the MEP surface are located on the hydrogen atoms which are expected to be favorable sites for nucleophilic attack. The most negative regions of the molecule were detected around the carbonyl oxygen O1, phenol oxygen O2, inside the phenyl and phenol rings making them dominant sites for an electrophilic attack. The carbonyl oxygen has a higher electron density (denser red color), suggesting that it plays an important role in the biological activity through hydrogen bonding with the target. 

### 2.6. Mulliken Charge Analysis

**Figure 6:**
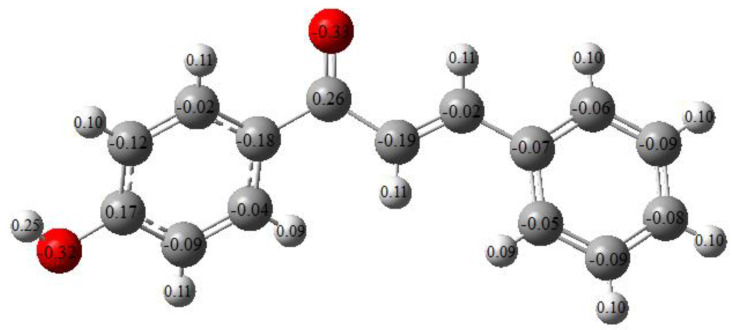
Mulliken charge distribution of the studied molecule.

The electronic charges have a crucial role in determining the bonding capability of a molecule. Mulliken charge values for the constituent atoms of the studied molecule are presented in Figure 6. The hydrogen atoms are all positively charged. The hydrogen atom adjacent to the oxygen atom has the highest positive charge. This is due to the electronegative nature of oxygen atoms. Similarly, the charges of the two carbons bonded to oxygen atoms were found to be positive among the rest of the negatively charged carbon atoms.

### 2.7. Molecular Docking

**Figure 7:**
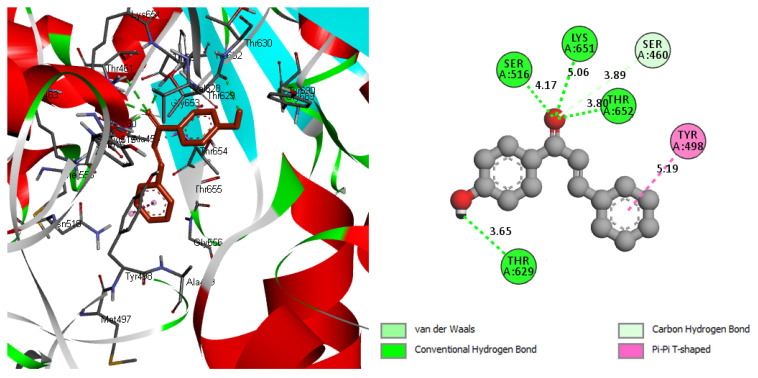
The binding interactions of 1-(4-Hydroxyphenyl)-3-phenylprop-2-en-1-one ligand with PBP 1 of S. aureus protein.

The molecular docking information was listed in Table 4. The binding energy was found to be −7.40 Kcal/mol. The achieved result counts as the lowest stable binding affinity compared to a docking study with similar chalcone structures against S. aureus, the best binding mode obtained in this study was −6.30 Kcal/mol [37]. The calculated low value of the inhibition constant of 3.74 µM compared to the mentioned literature indicated a high inhibition ability for the studied molecule. The docked sites of the ligand with a pocket cavity of the target protein involved in six interactions are shown in Figure 7 (see Supplementary Materials). The formation of hydrogen bonds is documented as a sign of high binding affinity [38]. The carbonyl group contributed with a vital role in the high inhibition capability of the ligand by forming three conventional hydrogen bonds (THR625 → 3.80 Å, LYS651 → 5.06 Å, and SER516 → 4.17 Å) and van der Waals bond with SER460 → 3.89 Å. Another hydrogen bond has been formed by an oxygen atom in phenol with THR629 amino acid. The binding interactions also led to the T-shaped π–π interaction between the phenyl ring and TYR498, which contributed to stabilizing the binding mode for the protein-ligand complex. Although the investigated ligand had s.cis-configuration, the docking interaction induced the form of the ligand to be s.trans as shown in Figure 7. This chalcone reaction suggests that trans-chalcone is a thermodynamically favorable form in polar solvents in the same environment as the solvated docking interaction [39].

## 3. Materials and Methods

### 3.1. Experimental Details

The synthesis of 1-(4-Hydroxyphenyl)-3-phenylprop-2-en-1-one molecule was previously reported by V. Parthasarathi and et al. [31]. The data collection and geometrical parameters of the described compound were included in this study. FTIR and FT-Raman spectra were collected from the spectra base of Wiley Science Solutions (Spectra Base-Wiley Registry Know-it-all Spectral Libraries) [40]. The scale range of the FT-IR spectrum was 500 to 3500 cm^−1^ while that of the FT-Raman spectrum was 300 to 3100 cm^−1^.

### 3.2. Computational Methods

DFT expressed in B3LYP/6-311G** model was used to calculate the ground-state molecular structure of the title molecule. B3LYP is an acronym for Becke’s three-parameter exchange function in combination with Lee–Yang–Parr nonlocal correlation functional and is commonly used in quantum chemical calculations for its reliable results [41,42]. The experimental single-crystal X-ray analysis was used to generate the initial molecular geometry of the studied molecule. The vibrational frequency results were obtained from the optimized structural parameters by using the same level of calculations (B3LYP method with 6-311G** basis set). All calculations were accomplished with the Gaussian 09W program [43]. The graphical interface Gauss View 6 was used to visualize the optimized geometry, HOMO-LUMO, and MEP as well as the assignments of the vibrational bands [44]. 

There are various numbers of penicillin-binding proteins (PBPs) that are housed in the outer cell wall of the bacteria. These sites of PBPs are classified as excellent targets for inhibitor drugs of bacterial cells as facilitating the ligand penetration [45,46]. Round-shaped bacteria known as Staphylococcus aureus (S. aureus) is one of the most common causes of mortality and morbidity associated with public society and hospitals. S. aureus includes four components of PBPs (symbolized as PBP 1-4) in methicillin-susceptible Staphylococcus aureus strains. PBP1 is the key protein with transpeptidase (ATPase) activity and has an essential role in cell division and separation in S. aureus [47,48]. Therefore, PBP1 was chosen as the target protein to be docked with our studied molecule. The crystallographic structure of the penicillin-binding protein (PBP-1b) of S. aureus was retrieved from the Protein Data Bank (PDB code: 2Y2H) and was implemented as the target protein. The optimized structure from DFT calculations was used as the initial ligand of the docking study. Auto Dock Tools 1.5.6 was utilized for the simulation of the docking interaction between the optimized molecule and the target protein [49]. The resulting complex of ligand-protein interaction was explored by using Discovery Studio 4.5 Software [50].

## 4. Conclusions

In this work, DFT calculations using B3LYP/6-311G** computational model were performed for 1-(4-Hydroxyphenyl)-3-phenylprop-2-en-1-one molecule. The optimized structural parameters including the bond lengths and bond angles were determined and compared with the previous experimental results. The calculated vibrational wavenumbers of the FT-IR and Raman spectra agreed well with data obtained from the experimental study. The calculated values of some quantum chemical parameters confirmed the high chemical reactivity of the molecule. MEP and Mulliken charge results were used to identify the most reactive sites. The molecular docking has been utilized to predict the binding of the title molecule within the active site of penicillin-binding proteins PBP1, the key protein of cell division and separation in S. aureus bacteria. The carbonyl group contained in the molecule exhibited high binding affinity through three H-bonds formed with the receptor protein. The minimum binding energy and the small value of the inhibition constant in the obtained results revealed the high inhibition efficiency of the studied molecule against S. aureus bacteria.

## Figures and Tables

**Table 1:** Selected calculated and experimental FT-IR vibrational frequencies for 1-(4-Hydroxyphenyl)-3-phenylprop-2-en-1-one.

	Observed Frequencies		Calculated	Frequencies	Assignment
No	FT-IR	FT Raman	Unscaled	Scaled	
1	⎯⎯	⎯⎯	3825	3787	ν (O–H)
2	⎯⎯	⎯⎯	3212	3179	ν (C–H)
3	⎯⎯	⎯⎯	3203	3170	ν (C–H)
4	⎯⎯	⎯⎯	3197	3165	ν (C–H)
5	⎯⎯	⎯⎯	3193	3161	ν (C–H) R s
7	3120	3060	3184	3152	ν (C–H)
8	⎯⎯	⎯⎯	3177	3145	ν (C–H) R as
9	⎯⎯	⎯⎯	3163	3131	ν (C–H) R as
10	⎯⎯	⎯⎯	3150	3118	ν (C–H)
11	⎯⎯	⎯⎯	3149	3117	ν (C–H)
12	1648	1645	1723	1705	ν (C=O)
13	1607	1594	1654	1637	ν (C=C)
14	1594	⎯⎯	1645	1628	ν (C=C) + ν (C=O)
15	1574	1552	1637	1620	ν (C=C) + ν (C=O)
16	1569	⎯⎯	1623	1606	ν (C–H) R as
17	1551	⎯⎯	1615	1599	ν (C–H) R s
18	1333	1319	1364	1350	γ (C–H)R
19	⎯⎯	⎯⎯	1321	1308	γ (C–H)
20	1281	⎯⎯	1301	1287	ν (C–O)
21	1165	1198	1188	1176	α(C–H)
22	⎯⎯	⎯⎯	1184	1172	α(C–H)
23	⎯⎯	⎯⎯	1130	1118	α(C–H)
24	⎯⎯	⎯⎯	1104	1093	α(C–H)
25	978	998	994	984	twist (C–H)
26	⎯⎯	⎯⎯	980	970	twist (C–H)
27	⎯⎯	⎯⎯	950	940	twist (C–H)
28	⎯⎯	⎯⎯	931	921	twist (C–H)
29	772	⎯⎯	784	776	ω (C–H)
30	⎯⎯	⎯⎯	744	737	ω (C–H) as+ (C=C)
31	⎯⎯	⎯⎯	705	698	ω (C–H) R

R—Ring, s—symmetric, a—asymmetric, ν—stretching, γ—rocking, α—scissoring, twist: twisting, ω—wagging.

**Table 2:** The calculated and experimental values of selected structural parameters, bond lengths (Å), and bond angles (°) of 1-(4-Hydroxyphenyl)-3-phenylprop-2-en-1-one.

Bond Length (Å)	Exp.	B3LYB/6-311g(d,p)	Bond Length (Å)	Exp.	B3LYB/6-311g(d,p)
C_9_-O_1_	1.24	1.23	C_7_-C_8_	1.32	1.34
C_13_-O_2_	1.34	1.36	C_8_-C_9_	1.47	1.48
C_1_-C_2_	1.38	1.39	C_9_-C_10_	1.46	1.49
C_2_-C_3_	1.36	1.39	C_10_-C_11_	1.39	1.40
C_3_-C_4_	1.36	1.39	C_11_-C_12_	1.37	1.38
C_4_-C_5_	1.38	1.38	C_12_-C_13_	1.39	1.39
C_6_-C_1_	1.38	1.40	C_13_-C_14_	1.38	1.40
C_5_-C_6_	1.38	1.40	C_15_-C_14_	1.37	1.38
C_6_-C_7_	1.46	1.46	C_10_-C_15_	1.39	1.40
**Angle (◦)**	**Exp.**	**B3LYB** **/6-311g(d,p)**	**Angle (◦)**	**Exp.**	**B3LYB** **/6-311g(d,p)**
C_13_-O_2_-H	109.5	109.40	C_10_-C_9_-C_8_	118.68	119.23
C_11_-C_10_-C_15_	117.61	117.97	C_12_-C_11_-C_10_	121.63	121.30
C_11_-C_10_-C_9_	122.0	124.56	C_14_-C_15_-C_10_	121.2	121.31
C_15_-C_10_-C_9_	120.39	117.44	O_2_-C_13_-C_14_	122.98	122.67
C_5_-C_6_-C_1_	117.90	118.03	O_2_-C_13_-C_12_	117.64	117.54
C_5_-C_6_-C_7_	122.47	123.45	C_8_-C_7_-C_6_	126.6	124.95
C1-C6-C7	119.70	118.51	C_8_-C_7_-H_7_	116.7	115.23
O_1_-C_9_-C_10_	121.17	119.95	C_7_-C_8_-C_9_	122.4	121.9
O_1_-C_9_-C_8_	120.15	120.83	C_6_-C_1_-C_2_	121	120.87
C_6_-C_5_-C_4_	120.90	120.12	C_3_-C_2_-C_1_	119.9	117.35
C_14_-C_13_-C_12_	119.40	118.55	C_15_-C_14_-C_13_	120.14	119.27
C_11_-C_12_-C_13_	120	119.80	C_4_-C_3_-C_2_	120.2	119.86
C_7_-C_8_-C_9_-O_1_	−11.40	−11.37	C1-C6-C5-C4	−1.2	−1.19
C_10_-C_9_-C_8_-C_7_	168.50	167.42	C_7_-C_6_-C_5_-C_4_	177.1	175.54
C_5_-C_6_-C_1_-C_2_	1.5	1.31	C_6_-C_5_-C_4_-C_3_	0.7	0.68
C_5_-C_6_-C_7_-C_8_	−7.8	−7.2	C_6_-C_1_-C_2_-C_3_	−1.2	−1.17
C_7_-C_6_-C_1_-C_2_	−176.9	−175.66	C_6_-C_7_-C_8_-C_9_	−177.17	−176.58
C_1_-C_6_-C_7_-C_8_	170.5	169.42	C_9_-C_10_-C_11_-C_12_	−179.4	−178.32
C_12_-C_11_-C_10_-C_9_	−179.4	−178.01	C_11_-C_10_-C_15_-C_14_	0.8	0.78
C_9_-C_10_-C_15_-C_14_	−179.15	−177.89	C_10_-C_15_-C_14_-C_13_	−1.7	−1.67
C_11_-C_12_-C_13_-O_2_	−179.3	−177.53	C_12_-C_13_-C_14_-C_15_	1.2	1.17
C_15_-C_14_-C_13_-O_2_	−179.4	−179.11	C_10_-C_11_-C_12_-C_13_	−1.1	−1.15
C_11_-C_10_-C_9_-O_1_	155.7	153.64	C_14_-C_13_-C_12_-C_11_	0.2	0.17
C_15_-C_10_-C_9_-O_1_	−24.4	−23.05	C_15_-C_10_-C_9_-C_8_	155.73	154.22
C_11_-C_10_-C_9_-C_8_	−24.2	−25.21	C_15_-C_10_-C_11_-C_12_	0.6	0.56

**Table 3:** Calculated quantum chemical parameters for the studied molecule; HOMO & LUMO energies, chemical hardness, chemical softness, electronegativity, and electrophilicity index.

Molecular Property	E_HOMO_ (eV)	E_LUMO_ (eV)	η (eV)	S (eV) ^−1^	χ (eV)	ω (eV)	N (eV)
**Definition**	⎯⎯	⎯⎯	$E_{L}-E_{H}$	$\frac{1}{\eta }$	$\frac{-\left(E_{H}+E_{L}\right)}{2}$	$\frac{\chi^{2}}{2\eta }$	$\frac{E_{H}-E_{H\left(\mathrm{TCE}\right)}}{2}$
**Value**	−6.42	−2.24	4.18	0.24	4.33	2.25	2.95

**Table 4:** The binding energy and interacted residues obtained from molecular docking calculations.

	Binding Energy (kcal/mol)	Inhibition Constant Ki (µM)	Intermolecular Energy (kcal/mol)	Bonded Residues
**(PBP-1b) protein**	−7.40	3.74	−8.6	THR629, THR625 LYS651, SER516 SER460, TYR498

## Supplementary Materials

The following data are available online; DFT optimized structure file for 1-(4-Hydroxyphenyl) -3-phenylprop-2-en-1-one containing the X, Y, and Z Coordinates (Å), File of the best docking site for the protein-ligand interaction, including X, Y, and Z Coordinates (Å) for the protein as well as the ligand.

## Sample Availability

Samples of the compounds are not available from the authors.
